# First record and DNA barcoding of *Donacaulaniloticus* (Zeller, 1867) from the Iberian Peninsula (Lepidoptera: Crambidae)

**DOI:** 10.3897/BDJ.9.e70193

**Published:** 2021-11-16

**Authors:** Juan J Guerrero, Manuel Pozas, Antonio S Ortiz

**Affiliations:** 1 Universidad de Murcia, Murcia, Spain Universidad de Murcia Murcia Spain; 2 Bahía de Cádiz, 9, Rota, Spain Bahía de Cádiz, 9 Rota Spain

**Keywords:** taxonomy, occurrence, cytochrome oxidase, mitochondrial, Cádiz, Spain

## Abstract

**Background:**

*Donacaulaniloticus* (Zeller, 1867) is known from south-eastern Europe, Middle East and Turkey to Central Asia, northern India and China and widely distributed in North Africa (Morocco, Algeria, Libya and Egypt).

**New information:**

*Donacaulaniloticus* (Zeller 1867) is recorded for the first time from the Iberian Peninsula and the first DNA barcode sequence is published and compared with other European and North American *Donacaula* species.

## Introduction

The family Crambidae (Pyraloidea) currently includes 10,343 species in 15 subfamilies worldwide of which 239 species in 29 genera belong to the subfamily Schoenobiinae (Léger et al. 2020). Many species of Schoenobiinae occur in wetlands and have semi-aquatic larvae. Adults are often found resting on grasses, rushes or sedges and larvae of some species are known to be stem borers in grasses and sedges in hygrophilous Poaceae, Juncaceae and Cyperaceae (Ferguson 1975, Robinson et al. 2002, Speidel 2005, White et al. 2005). In Europe, Schoenobiinae is represented by six species: *Scirpophagapraelata* (*Scopoli 1763*), *S.xanthopygata* Schawerda 1922, *Schoenobiusgigantellus* ([Denis and Schiffermüller] 1775), *Donacaulaforficella* (Thunberg 1794), *D.mucronella* ([Denis and Schiffermüller] 1775) and *D.niloticus* (Zeller 1867).

*Donacaula Meyrick 1890* is known to occur in the Palearctic, Ethiopian, Oriental, Neotropical and Nearctic Regions, except in the Australian Region. The genus includes 31 described species, of which four occur in the Palearctic, four in the Ethiopian, two in the Oriental, nine in the Neotropical and twelve in the Nearctic Regions (Speidel 2005). Adults of *Donacaula* have high variability between individuals and between geographical populations with forewings that are light brown to yellowish-brown with some species having dark longitudinal lines and/or spots and apices of forewings can be pointed in females, but are always rounded in males (Speidel 2005).

*Donacaulaniloticus* is known from south-eastern Europe, Middle East and Turkey to Central Asia, northern India and China and widely distributed in North Africa from Morocco to Egypt (Speidel 2005, Slamka 2008, Leraut 2012). In the Iberian Peninsula, two species of *Doncaula* have been recorded, *D.forficella* and *D.mucronella* according to Speidel (2005) and Vives-Moreno (2014), although Slamka (2008) and Leraut (2012) considered that *D.mucronella* is not present in the Iberian Peninsula. In relation to the biology of *D.niloticus*, the early stages are unknown, whilst the adults fly in several generations from April to September in riparian and steppe vegetation with nearby watercourses and lakes (Speidel 2005, Slamka 2008, Leraut 2012).

In this article, we provide the first record and DNA barcoding of *Donacaulaniloticus* (Zeller, 1867) collected from the saltmarshes of Adventus in Trebujena (Cádiz, Spain). The specimens collected have some consistent morphological features which differ from the other *Donacaula* species. In addition, mtDNA sequence(COI) were used to assess genetic divergence between the *Donacaula* species from Europe and North America.

## Materials and methods

### Morphological study

**Figure 1.**
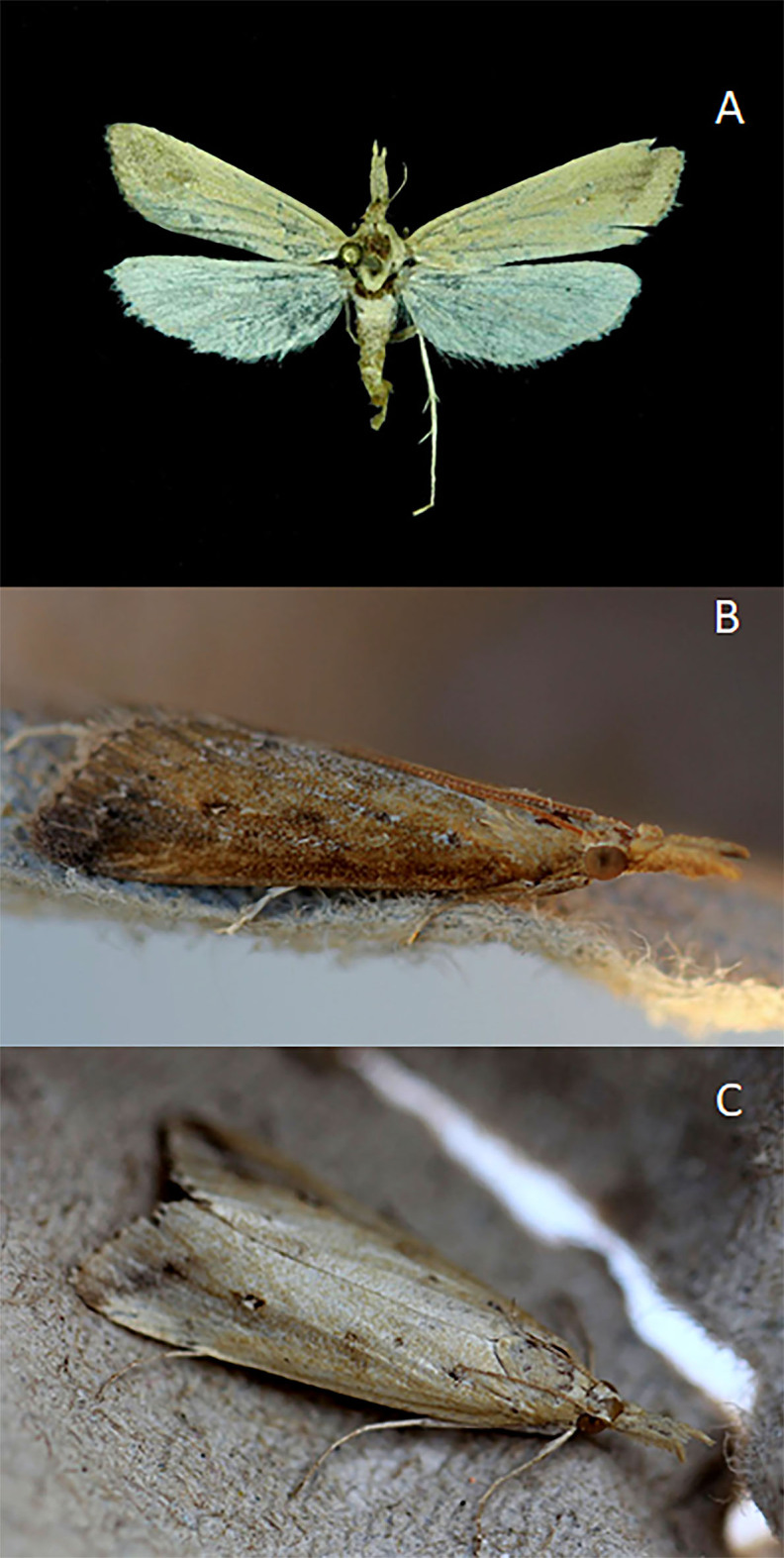
Adult specimens of *Donacaulaniloticus* (Zeller) from Marismas de Trebujena, Cádiz, 8-IX-2020. **A**. Female specimen (A.S. Ortiz); **B.** Male specimen photographed at the same locality and date (M. Pozas); **C.** Female specimen photographed at the same locality and date (M. Pozas).

During the sampling, one female specimen with a white discocellular spot and unpointed apex traits was collected (Fig. 1a). Additionally, one male with a dark brownish line from the costal margin to about the centre of the wing was photographed (Fig. 1b and c).

The specimen was pinned, wings spread and dried. Their external characters were examined in order to evaluate possible differences in colouration and wing shape. Furthermore, it was dissected using standard procedures (Hausmann 2001) with minor modifications. The female adult image was taken with a Nikon D70 digital camera. Images were z-stacked using Zerene software. The female genitalia were studied using a Zeiss Stemi 508 stereomicroscope with a Zeiss Axiocam ICc5 digital camera. The specimen is deposited in the Research Collection of Animal Biology (RCBA-UMU) in the Department of Zoology and Physical Anthropology of the University of Murcia (Spain).

### Molecular study

For DNA extraction, two legs were removed from the specimen in order to sequence the 658 base-pair long barcode segment of the mitochondrial COI gene (cytochrome c oxidase 1, 5’ terminus). The tissue samples were submitted to the Canadian Centre for DNA Barcoding (CCDB, Biodiversity Institute of Ontario, University of Guelph) to obtain DNA barcodes using the high-throughput protocol described in deWaard et al. (2008) which can be accessed at www.dnabarcoding.ca/pa/ge/research/protocols. The DNA extracts are currently stored at the CCDB and the sequences are deposited in GenBank according to the iBOL data release policy (MZ920225).

Voucher data, images, sequences and trace files are publicly available on the Barcode of Life Data System (BOLD) (Ratnasingham and Hebert 2007). Sequence divergences for the barcode region were calculated using the Kimura 2-parameter (K2P) model and the degrees of interspecific genetic variation were calculated using the analytical tools of BOLD. All of the new and related species sequences were downloaded and aligned with the CLUSTAL algorithm of the MEGA6 software (Tamura et al. 2013), including all sites with the pairwise deletion option. A phylogenetic hypothesis with Maximum Likelihood as an optimality criterion was generated using IQ-TREE v.1.6.12 (Nguyen et al. 2015). An alignment of 658 bps for 99 samples was partitioned into codon positions with ModelFinder software (Kalyaanamoorthy et al. 2017) and the 1st codon position was modelled with TN+F+G4; 2nd with TIM3e+G4; 3rd with TPM3u+F+I. Support values were calculated by 1,000 replications of both ultrafast bootstrap (UFBoot; Hogan et al. 2017) and Shimodaira-Hasegawa-like approximate likelihood ratio test (SH-aLRT; Guindon et al. 2010), as well as approximate Bayes branch test (aBayes; Anisimova et al. 2011). In order to assess the COI divergences between *Donacaulaniloticus* and the *other Donacaula* species from Europe and from North America, we included all sites with the pairwise deletion option. The public sequences of *Donacaulaforficella* (BOLD:AAC9877; n = 24 seqs), *D.mucronella* (BOLD:AAE8467; n = 21) from Europe and *D.longirostrallus* (Clemens 1860) (BOLD:AAF5260; n = 13), *D.maximellus* (Fernald 1891) (BOLD:AAE8462; n = 1), *D.melinellus* (Clemens 1860) (BOLD:AAB7736; n = 19), *D.roscidellus* (Dyar 1917) (BOLD:AAD3103; n = 8), *D.sordidellus* (Zincken 1821) (BOLD:AAE8462; n = 1) and *D.unipunctellus* (Robinson 1870) (BOLD:AAA4113; n = 11) from North America were obtained from the public database in BOLD. We selected *Scirpophagapraelata* (BOLD:AAB6864; n = 1), which is systematically related into subfamily Schoenobiinae as outgroup to root the trees.

## Taxon treatments

### Donacaula niloticus

(Zeller, 1867)

BA57C87D-617C-568C-BEB0-08E4550B3C75

Schoenobius niloticus Zeller 1867: 462, pl. 24, fig. 2.
(TL: Egypt, Alexandria)

#### Materials

- **Type status:** Other material. **Occurrence:** recordedBy: J.J. Guerrero; individualCount: 1; sex: female; lifeStage: adult; disposition: in collection; **Taxon:** scientificName: Donacaulaniloticus (Zeller, 1867); kingdom: Animalia; phylum: Arthropoda; class: Insecta; order: Lepidoptera; family: Crambidae; genus: Donacaula; specificEpithet: niloticus; taxonRank: species; verbatimTaxonRank: sp.; scientificNameAuthorship: (Zeller, 1867); taxonomicStatus: accepted; **Location:** country: Spain; stateProvince: Andalusia; county: Cadiz; locality: Trebujena, Saltmarshes of Adventus, Seno de la Esparraguera; verbatimCoordinates: 36°53'31.20"N 6°15'36.00"W; verbatimLatitude: 36°53'31.20"N; verbatimLongitude: 6°15'36.00"W; **Identification:** identifiedBy: J.J. Guerrero; dateIdentified: 2020; **Event:** samplingProtocol: light trap; eventDate: 08/09/2020; year: 2020; month: 9; day: 8; **Record Level:** institutionCode: ZAF-UMU; collectionCode: RCBA; basisOfRecord: PreservedSpecimen

#### Taxon discussion

**Figure 2.**
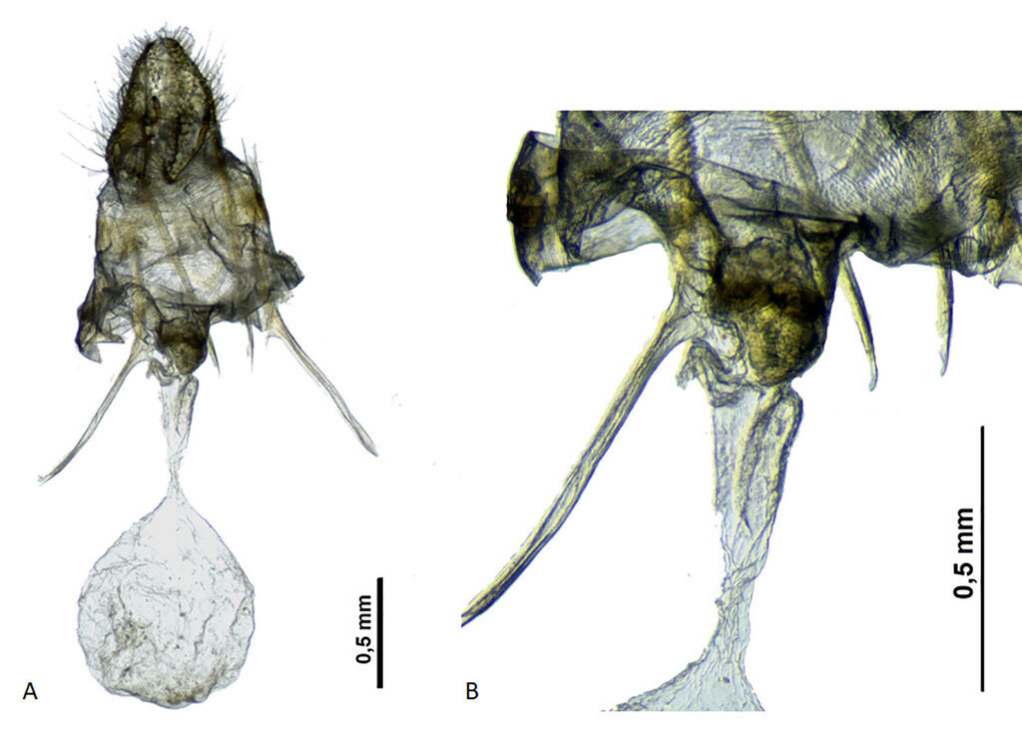
Female genitalia of *Donacaulaniloticus* (Zeller). Genital prep. slide G700. **A.** General view ; **B.** Ductus bursae detail.

The Iberian specimen showed morphological traits (Figs 1, 2) typical of European individuals of *Donacaulaniloticus* according to the diagnosis in Goater et al. (2005), Slamka (2008)and Leraut (2012). These diagnostic characters, compared to the other European species of *Donacaula*, are for the male: forewing with dark brownish line from the costal margin to approximately the centre of the wing, parallel to the outer margin; and for the female: forewing with white discocellular spot and apex non-pointed. Integrating the evidence from COI mitochondrial DNA sequences and adult morphology, we conclude that the *D.niloticus* specimen collected in the saltmarshes of Adventus (Cádiz) is genetically different to other species included in the genus *Donacaula* from Europe and North America.

## Analysis

The COI divergences between *Donacaulaniloticus* and seven *Donacaula* species from Europe and North America, including *Scirpophagapraelata* as additional Schoenobiinae species, are presented in Table 1.

All trees presented the same topology and were practically identical; therefore, only the ML tree is presented here with the branch tips collapsed since each of the groups of sequences correspond to a single BIN (Fig. 4). The complete tree can be consulted at Suppl. material 1. As one gene is insufficient for reasonable phylogenetic analysis (Gatesy et al. 2007), the tree presented here does not reliably illustrate evolutionary relationships amongst the sequenced taxa.

## Discussion

The presence of *Donacaulaniloticus* in the riparian vegetation near to the mouth of the Guadalquivir River into the saltmarshes of Adventus (Trebujena, Cádiz) is confirmed by integrating the evidence from COI mitochondrial DNA sequences and adult morphology and is genetically different to the other two European and six North American *Donacaula* species. Molecular data indicate significant divergence with large mean distances amongst *Donacaula* species (13.0%) with maximum distance between *D.sordidellus* and *D.longirostrallus* and minimum distance between *D.melinellus* and *D.mucronella* (7.6%). In the case of *D.niloticus*, the distance to the European *Donacaula* species was 13% to *D.forficella* and 11.8% to *D.mucronella*, while the genetic divergence amongst *D.forficella* and *D.mucronella* was 13%. (Table 1, Fig. 4). Although the genetic differences between all the species of *Donacaula* are similar, the topology of the tree relates them and separates the other species of Schoenobiinae (*Scirpophagapraelata*).

**Figure 3.**
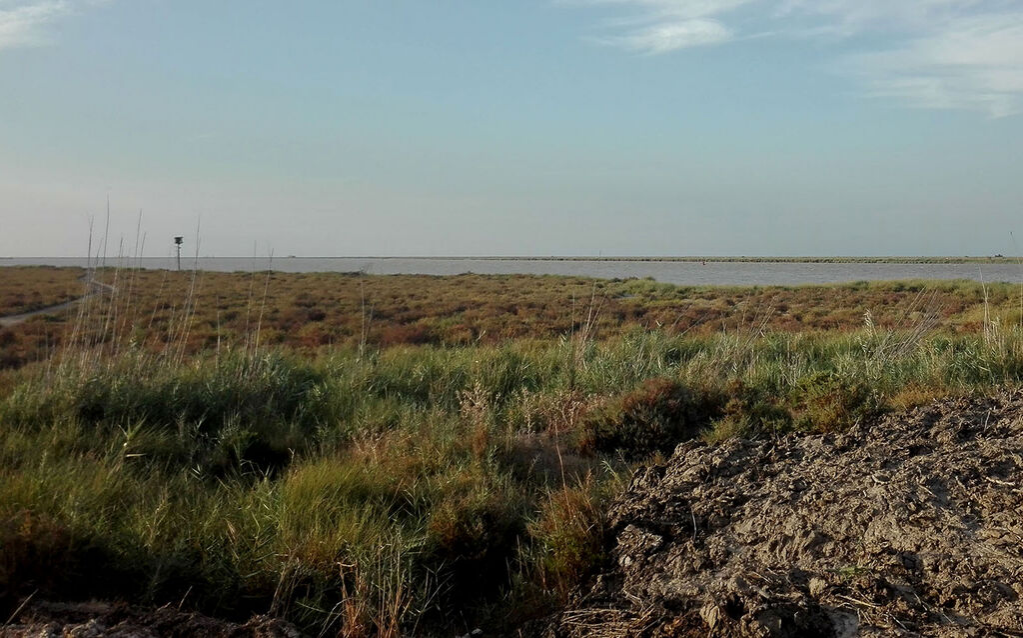
Habitat in the saltmarshes of Adventus near Trebujena, Cádiz (M. Pozas).

**Figure 4.**
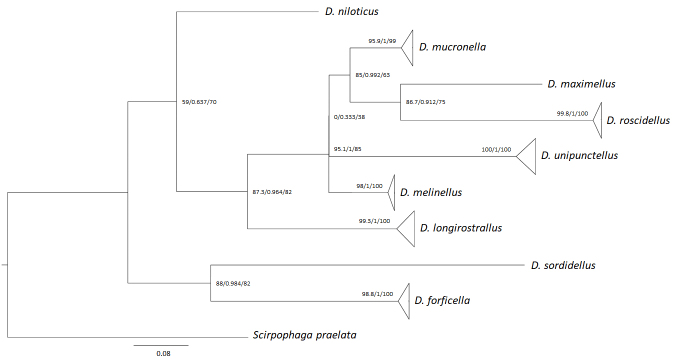
Maximum Likelihood tree (ML) (ML; constructed with IQ-TREE; COI 658 bp) including 99 sequences of selected *Donacaula* species rooted with *Scirpophagapraelata*. Branch supports are represented by SH-aLRT/aBayes/UFBoot.

*Donacaula* is associated with various semi-aquatic and marsh plants where their larval stages live in stems or roots or on exposed and non-submerged leaves of *Phragmitesaustralis*, *Glyceria* spp. and *Carex* spp. These lepidopterans have developed various strategies and adaptations that have allowed them to stay in close proximity to water (Pabis 2018). *D.niloticus* larvae in the lower part of stems and their presence is indicated by a small round hole in the stem five or eight centimetres above the root. Pupation take place in the base of the stem (Speidel 2005). The landscapes of the riverside plain of the Guadalquivir river, in the furthest point south of the Iberian Peninsula where *D.niloticus* was collected, are characterised by the irregular regime of water inputs, which can go from flooding the marshland to turning it into a desert dryland. These circumstances and the marine influence, due to its proximity to the river mouth, produce soils with varied salinity content that characterise the vegetation. This habitat is characterised by a series of hyperhalophilic Mediterranean-Ibero-Atlantic thermomediterranean edaphohygrophilic vegetation (EH20) which forms estuaries, salt marshes and marshes with a mixture of salty and fresh waters which is represented by the communities *Spartinetum maritimae, Puccinellio-Sarcocornietum perennis, Halimiono-Sarcocornietum alpini, Cistancho-Arthrocnemetum macrostachyi, Polygono-Limoniastretum monopetali* and, on the edge of the estuaries, the halonitrophilic community, *Cistancho-Suaedetumverae*. Sometimes, a plantation of *Polygono-Tamaricetumafricanae* may appear. The land use bordering the habitat are mainly agricultural fields (Fig. 3) (CMAOT 2015).

We emphasise here the importance of combining traditional morphological analysis and ecological traits with the additional dataset of DNA sequences for those taxonomic groups whose identification is particularly difficult and mainly based on differences in the genitalia.

## Supplementary Material

XML Treatment for Donacaula niloticus

A913552E-85EF-5492-9BCD-BBD720C494F6 10.3897/BDJ.9.e70193.suppl1 Supplementary material 1 Maximum Likelihood tree (ML) of the *Donacaula* genus, based on 99 sequences of the mtDNA COI gene Data type Maximum Likelihood tree (ML) Brief description Maximum Likelihood tree (ML) (ML; constructed with IQ-TREE; COI 658 bp) including 99 sequences of selected *Donacaula* species rooted with *Scirpophagapraelata*. Branch supports are represented by SH-aLRT/aBayes/UFBoot. File: oo_607849.pdf https://binary.pensoft.net/file/607849 Antonio S. Ortiz

## Figures and Tables

**Table 1.** Interspecific mean K2P (Kimura 2-Parameter) divergences (mean pairwise distances) between *Donacaula* species, based on the analysis of COI fragments (> 600 bp).

	*D.longirostrallus*	*D.melinellus*	*D.forficella*	*D.mucronella*	*D.roscidellus*	*D.unipunctellus*	*D.niloticus*	*D.sordidellus*	*Scirpophagapraelata*
*D.maximellus*	12.9	10.8	14.3	10.9	12.9	11.3	12.9	14.3	15.9
*D.longirostrallus*		12.2	14.7	12.5	15.3	14.9	13.2	15.6	16.2
*D.melinellus*			14	7.6	12.2	11.4	11.8	13.4	16.5
*D.forficella*				13	15.1	14	13	13.2	14.9
*D.mucronella*					12.5	11.9	11.8	14.1	15.2
*D.roscidellus*						13.4	13.6	14.9	16.1
*D.unipunctellus*							12.9	14.4	16.4
*D.niloticus*								12.8	15
*D.sordidellus*									16.1
