# Supplementary figures and images for: First record and DNA barcoding of Donacaulaniloticus (Zeller, 1867) from the Iberian Peninsula (Lepidoptera: Crambidae)
Source: Biodivers Data J. 2021 Nov 16;9:e70193. doi: 10.3897/BDJ.9.e70193 (PMC9878576; doi:10.3897/BDJ.9.e70193)

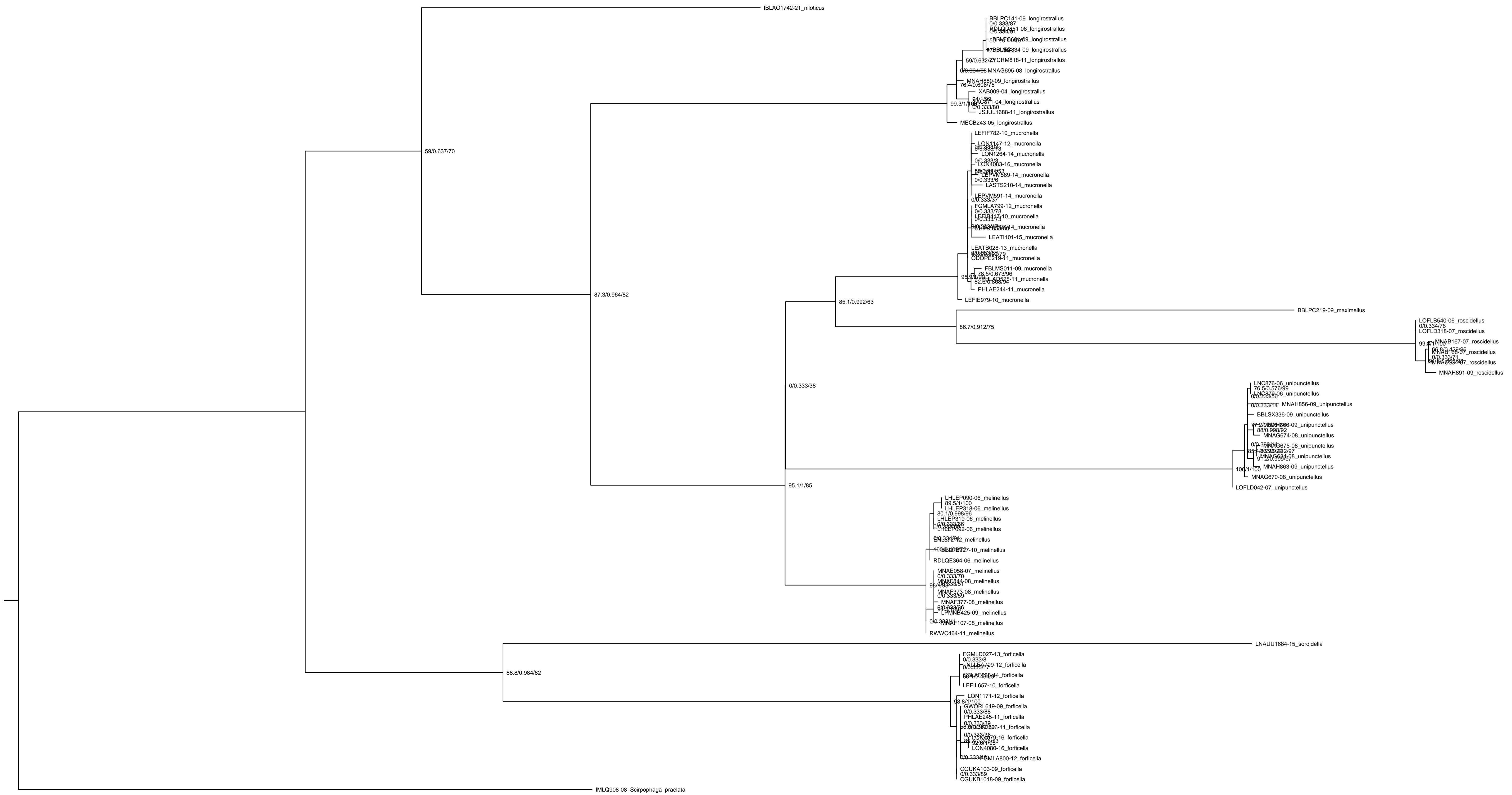

Supplement: Supplementary material 1 — Maximum Likelihood tree (ML) of the Donacaula genus, based on 99 sequences of the mtDNA COI gene [file bdj-09-e70193-s001.pdf]
